# Culm height development, biomass accumulation and carbon storage in an initial growth stage for a fast-growing moso bamboo (*Phyllostachy pubescens*)

**DOI:** 10.1186/s40529-016-0126-x

**Published:** 2016-04-28

**Authors:** Tian-Ming Yen

**Affiliations:** Department of Forestry, National Chung-Hsing University, 145 Xingda Road, South District, Taichung, 40227 Taiwan

**Keywords:** Carbon storage, Moso bamboo (*Phyllostachys pubescens*), The Richards growth function, Allometric model, Biomass

## Abstract

**Background:**

The purpose of this study was to predict culm height development and to evaluate biomass accumulation and carbon storage in the initial growth stage of moso bamboos (*Phyllostachys pubescens*). A total of 30 bamboos were sampled based on their diameter at breast height (DBH). I predicted the culm heights daily based on the Richards growth function for these bamboo samples. After they reached their maximum heights, the biomass and carbon storage were determined.

**Results:**

The results showed that the Richards function accurately simulated the height growth of bamboos and that the growth potential of culm heights increased with increasing DBH classes. In contrast, the time when the maximum growth rate occurred (*t*_*max*_) appeared to not be influenced by DBH classes and was close to 20 days for all DBH classes. The culms arrived at their maximum heights in about 40 days regardless of DBH class. In addition, astonishing biomass accumulation and carbon storage was found during this period, and the aboveground biomass and carbon storage were predicted to be 3.44–17.33 and 1.58–8.04 kg culm^−1^ for moso bamboos, respectively. The allometric model was used to predict the relationships between DBH and aboveground biomass in this stage.

**Conclusions:**

I compared the biomass accumulation between this stage and the entire yield period (5 years) and found that the bamboos accumulated three-fourths of their biomass for the entire yield period in only 40 days. This revealed that biomass accumulation and carbon storage mainly occurs in the initial growth stage for individual moso bamboos.

## Background

Special properties and growth patterns have been discovered in bamboo plants, which have grass-like leaves and a woody vascular bundle structure but belong to a particular taxonomic group of the grasses family (Poaceae) (Shanmughavel et al. 2001; Lu 2001; Yen et al. 2003a, b, 2010; Yen and Lee 2011; Yen 2015). Most bamboo is distributed in Asia (over 80 % of the world’s total), and according to estimates, more than 1000 species cover an area of over 180,000 km^2^ in Asia (Scurlock et al. 2000; Lu 2001; Embaye et al. 2005; Gratani et al. 2008; Yen et al. 2010). Thus, bamboos are common and important resources that have multiple uses for Asians, including uses as food and raw material (Lu 2001; Yen 2003; Yang et al. 2004; Yen et al. 2003a, b, 2010; Yen and Lee 2011; Yen 2015).

Since the signing of the Kyoto Protocol, the capability of carbon storage in woody plants has been widely examined (Lamolom and Savidge 2003; Yen et al. 2009, 2010; Yen and Lee 2011). In previous studies, an astonishing level of biomass accumulation in a short period was reported in some bamboos species, such as *Bambusa bambos* (Shanmughavel and Francis 1996), *Phyllostachys pubescens* (Isagi et al. 1997), makino bamboo (*Phyllostachys makinoi*) (Yen et al. 2010) and moso bamboo (*Phyllostachys pubescens*) (Yen and Lee 2011). Requiring only 5 years to grow from bamboo shoots to mature culms, the mature bamboo culms can then be harvested for use, indicating that bamboo is a superior species for carbon storage, especially under good management (Yen et al. 2010; Yen and Lee 2011; Yen 2015). Although bamboo culms cannot be harvested until they are 5 years old, biomass rapidly accumulates in the early growth stages (Yen et al. 2010; Yen and Lee 2011). In general, the development of bamboos can be classified into two obvious periods (Lu 2001; Yen 2003; Yen et al. 2010). The first stage is culm height growth (Yen 2003). Once they reach maximum height, the second stage begins, which is characterized by culms increasing in strength and accumulating dry mass until they are mature and can be harvested for human use (Lu 2001; Yen et al. 2010; Yen and Lee 2011). I found that many studies have focused on biomass accumulation and carbon storage during the second stage, but very few focus on the first stage (Shanmughavel et al. 2001; Yen et al. 2010; Yen and Lee 2011).

Bamboos grow rapidly during the first stage, indicating that biomass accumulates rapidly in bamboo bodies during this stage (Yen 2003). This study aimed to understand bamboo growth, biomass accumulation and carbon storage in the first stage for moso bamboo. I measured the heights of bamboo culms in different diameter classes daily, and then these bamboos were felled to determine their biomass and to estimate the carbon storage capacity after the culms reached maximum height. The growth patterns of culm heights were predicted by the Richards growth function to evaluate culm development for different DBH classes of moso bamboo. Our objectives were as follows: (1) to predict culm height growth based on the Richards growth function; (2) to compare growth patterns of culm heights between different DBH classes; and (3) to assess bamboo biomass and carbon storage during the first stage of growth for moso bamboo.

## Methods

### Study site

The study was conducted in moso bamboo forests located in central Taiwan (23°45′ N, 120°45′ E), at an elevation of 900–1200 m. This area is in the lower mountains and has abundant bamboo and timber resources, such as moso bamboo and China fir (*Cunninghamia lanceolata*) (Liu et al. 1988; Yen and Lee 2011). Moso bamboo is well known as it is one of the species with economic importance in Taiwan (Liu et al. 1988; Liu and Kao 1998; Lu 2001; Yen et al. 2003a, b; Yen and Lee 2011), namely for its culm, which can be used as a raw material, and its bamboo shoots, which are eaten by humans. In the study area, most moso bamboo forests are private, man-made plantations managed by farmers for their economic value, as well as being widely planted in general in this region (Yen and Lee 2011). Of these moso bamboo forests, four man-made plantations that belong to private farmers were used for this study. The major products of these bamboo forests address on shoot production because culm harvest is unprofitable in Taiwan. In general, most of private farmers target at shoot production which is common phenomenon for current bamboo forest management in Taiwan (Yen 2015). All four bamboo forests are under good management and have high productivity, with more than 20-year histories of management. That is, they are normally fertilized and thinned to maintain their productivity. Usually, this pattern is called intensive management that high cost and productivity are expected (Yen 2015). Therefore, these bamboo forests have a good stand structure, that is, 1- to 5-year-old culms have average distributions within the stands. Because these bamboo forests are distributed within the same region, a similar environment and climate can be obtained in the study region. The 4 bamboo plantations range in area from 1.34 to 2.15 ha. The region had a temperature range of 11–20.5 °C, a rainfall range of 1950–2350 mm year^−1^, and soil (brown humic and dark brown humic) with a depth of 40–95 cm.

### Research method

As previously mentioned, the 4 moso bamboo forests in this study are under good management, so they presented a good stand structure. As culms more than 5 years old should be harvested to maintain vigor and productivity, only 1 to 5-year-old culms are distributed throughout the stands. Moreover, the culm distributions are homogenous and regular within the bamboo forests. Six 0.01 ha (10 × 10 m) plots were randomly selected within each bamboo forest for 24 plots in total. The data were collected in 2007, and individual bamboos were selected for measuring culm height developments and predicting biomass and carbon storage during the initial growth stage.

For understanding the culm size distributions within the sample plots, the diameter at breast height (DBH) of all culms was measured in all sample plots before sampling individual bamboos to determine their height developments. The DBH distributions ranged mainly from 6–11 cm, and a stratified sampling method was proposed based on DBH. In general, this approach is wildly employed in bamboo studies because the bamboo samples can systemically distribute in each interval, especially using in predicting culm height, biomass and carbon storage (Yen 2003, 2015; Yen et al. 2010; Yen and Lee 2011). I adopted 1 cm as an interval in the present study, and sampled six bamboo plants in each 1 cm interval from 6–11 cm, for 30 total samples. However, the challenge is that future DBH development cannot be estimated for bamboo shoots emerging from the ground. Therefore, this study used triple samples (90 samples) as sample pools, and the culm heights were measured daily until reaching their maximum. Additionally, eight samples (or 8.89 % of the total samples) within the sample pools declined before they reached maximum height. I measured 30 samples from this sample pool based on the above-mentioned method (six samples within each DBH class, ranging from 6–11 cm), and then the plants were felled to determine the biomass of each component.

The culm height growth was obtained from the 30 sample bamboos based on daily records. Five DBH classes were classified, i.e., 6<DBH≦7; 7<DBH≦8; 8<DBH≦9; 9<DBH≦10 and 10<DBH≦11, and six bamboo samples within each DBH class were measured. The six bamboo samples within each DBH class were used as a basic data set. The relationships between culm heights and daily growth from each data set were used in the three-parameter Richards function to predict culm development. This growth function is widely used in bamboo culm growth, and is written as (Richards 1959; Yen 2003):

1 $$Y = A \times [1 - \exp ( - k \times t)]^{1/(1 - m)}$$

where *Y* is culm height, *t* is day, and *A*, *k*, and *m* are parameters of the Richards function.

Moreover, the differences in culm height growth between different DBH classes were compared and the relationships between the growth rate of culm height and time were determined via the Richards function.

To calculate the biomass, each bamboo sample was divided into 1 m sections to obtain the fresh weight of each component of foliage, branches and culms separately. Some of these components were sampled and weighed for each bamboo, and then they were taken to the laboratory to oven-dry at 105 °C (Yen and Lee 2011). After 2–3 weeks, the weight of each component was no longer decreasing, implying that the purely dry biomass of the foliage, branches and culms had been obtained. Moreover, the biomass of each component, i.e., the foliage, branches and culms, can be calculated based on the ratio of the absolute dry weight to the fresh weight of each section (foliage, branches, and culms). The relationships between aboveground biomass and DBH were predicted by a simple allometric function of Y = a × DBH^b^ (Y is aboveground biomass, and a and b are parameters). In addition, the allometric function of a 5-year-old plant in the same study area was cited to compare the contributions of biomass accumulation in the initial stage, where the function is Y = 0.017 × DBH^3.03^ (Yen and Lee 2011). The observed aboveground biomass data were also compared to the allometric function of the 5-year-old plant to calculate the contributions of biomass accumulation in the initial stage to the entire growth period (based on 5-year-old) for moso bamboo.

Moreover, the carbon storage of each component was calculated from the biomass × the percent carbon content (PCC). The PCC was determined for moso bamboo in central Taiwan by Wang et al. (2009), and I cited these PCCs as a base to predict carbon storage for each component of moso bamboo in this study. The PCCs of the foliage, branches and culms were predicted to be 45.44, 48.15 and 46.28 %, respectively, by Wang et al. (2009).

## Results

Before the individual bamboo samples were measured, the four moso bamboo forests were surveyed based on 24 sample plots. The stand characteristics of moso bamboo in culms per ha, mean DBH and aboveground biomass were 7188 ± 489 culms ha^−1^, 8.9 ± 1.1 cm and 81.6 ± 18.2 Mg ha^−1^, respectively. The aboveground biomass was predicted by the allometric model that was built by Yen and Lee (2011) in the same region.

The 30 bamboo samples were measured after the culms reached their maximum height, and the sample characteristics of mean DBH and culm height are shown in Table 1.

**Table 1:** The sample characteristics of mean diameter at breast height (DBH) and culm height in each diameter class

DBH class	Range (cm)	Sample number	DBH (cm)	Culm height (m)
I	6<DBH≦7	6	6.58 ± 0.26^a^	8.57 ± 0.57
II	7<DBH≦8	6	7.40 ± 0.28	9.53 ± 0.68
III	8<DBH≦9	6	8.48 ± 0.29	10.76 ± 0.94
IV	9<DBH≦10	6	9.55 ± 0.26	12.18 ± 0.72
V	10<DBH≦11	6	10.48 ± 0.25	12.83 ± 0.73

^a^Mean ± standard deviation

**Fig. 1:**
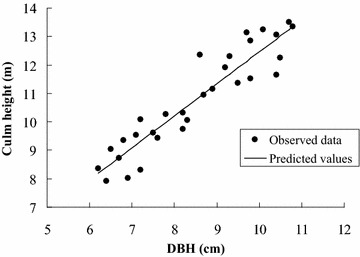
Relationships between DBH (*X*) and culm height (*Y*) for moso bamboo. A linear regression of *Y* = 1.22 + 1.12*X* (*R* = 0.93) is used to predict culm height by DBH

The culm height of bamboos increases with increasing DBH class (Table 1). Previous studies have shown a strong correlation between DBH and culm height for bamboos (Yen et al. 2010; Yen and Lee 2011). Using a correction analysis to examine the relationships between DBH and culm height, I found a strong positive correlation between these two variables (*R* = 0.93). Moreover, the DBH is taken as a dependent variable (*X*) to predict the culm height (*Y*), and a linear regression equation of *Y* = 1.22 + 1.12*X* (*R* = 0.93, *F* = 183, *P* = 0.000) is obtained. The relationships between the regression model and observed data are shown in Fig. 1.

Figure 1 shows a clear trend between DBH and culm height and a linear relationship between these two variables.

We used the three-parameter Richards equation to predict height growth of the culms daily, and the parameters of each age class are shown in Table 2. The Richards model fit the observed data with high *R* ^*2*^ (0.9602–0.9984) and small *RMSE* (0.1072–0.5368 m) for all DBH classes, indicating that this model accurately simulated the height growth of bamboos (Table 2). Moreover, a clear trend was found in the parameters of the Richards model among the DBH classes. The parameter ‘*A*’ increases with increasing DBH classes, while the parameters ‘*k*’ and ‘m’ appeared to remain constant for all DBH classes. This indicates that the *A* parameter was influenced by the DBH class, but the other two parameters were not (Table 2).

**Fig. 2:**
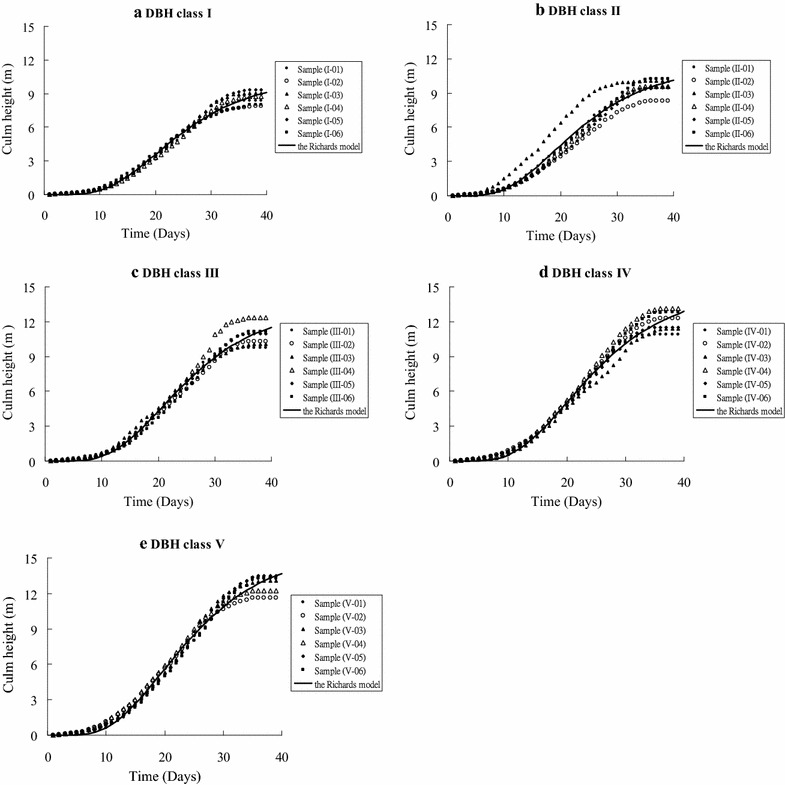
The actual culm height growth of the different DBH classes for moso bamboo and the predictions of the Richards model used to fit the observed data of each DBH class (where **a** DBH class I: 6<DBH≦7; **b** II: 7<DBH≦8; **c** III: 8<DBH≦9; **d** IV: 9<DBH≦10 and **e** V: 10<DBH≦11)

Using the observed data, I fit the Richards function (Table 2) for each DBH class of moso bamboo, as illustrated in Fig. 2. I found that the observed data of the growth patterns showed a sigmoid shape for bamboo height growth and that the observed data closely match the predicted curves, revealing that the Richards function accurately simulated bamboo height development.

**Table 2:** The parameters of the Richards function in each diameter class

DBH class	$Y = A \times [1 - \exp ( - k \times t)]^{1/(1 - m)}$				
	*a*	*k*	*m*	*R* ^*2*^	*RMSE* ^*a*^
I	10.46	0.0996	0.8634	0.9898	0.1072
II	11.40	0.1006	0.8490	0.9602	0.5368
III	13.40	0.0980	0.8664	0.9838	0.2725
IV	14.77	0.0993	0.8625	0.9873	0.2678
V	15.66	0.0987	0.8547	0.9914	0.2050

^a^ *RMSE* is the smallest root mean squared error and its unit is m

**Fig. 3:**
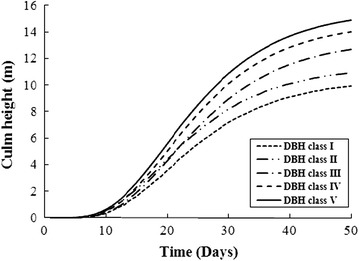
Development of culm height compared between the different DBH classes based on the Richards function for moso bamboo

The development of culm height was compared between different DBH classes based on the Richards function and is shown in Fig. 3.

**Fig. 4:**
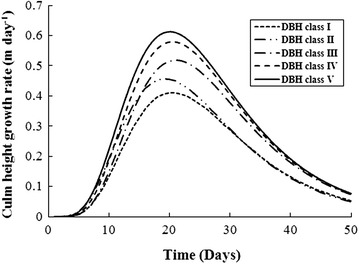
The growth rate of culm heights was calculated from the Richards function for the different DBH classes of moso bamboo

Comparing the culm height development between the different DBH classes, a regular growth pattern was discovered, namely a higher culm height with a larger DBH class at the same time in the growth process (Fig. 3). In addition, the growth rate of the culm height was calculated from the Richards function and is shown in Fig. 4.

Figure 4 reveals that the maximum growth rate (MGR) increases with increasing DBH classes while the time that the MGR occurred (*t*_*max*_) appears to be the same, at approximately 20 days, regardless of DBH class for moso bamboo (Fig. 4).

**Fig. 5:**
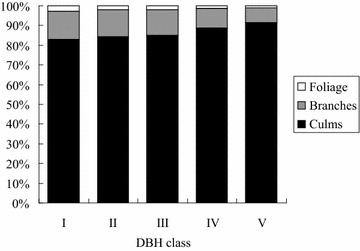
The proportion of foliage, branches and culms to total aboveground biomass in different DBH classes for moso bamboo in the initial stage of growth (where DBH class I: 6<DBH≦7; II: 7<DBH≦8; III: 8<DBH≦9; IV: 9<DBH≦10 and V: 10<DBH≦11)

The biomass accumulation and carbon storage of the different components was determined from 30 bamboo samples in the initial stage, and the means of these items are shown in Table 3. Moreover, the proportion of each component (foliage, branches and culms) to total aboveground biomass was taken into account for different DBH classes and is shown in Fig. 5.

**Table 3:** Average biomass accumulation and carbon storage in each diameter class for moso bamboo

Item	DBH class	Component			
		Foliage	Branches	Culms	Aboveground
Biomass (kg culm^−1^)	I	0.09 ± 0.03^a^	0.49 ± 0.14	2.82 ± 0.43	3.40 ± 0.50
	II	0.11 ± 0.04	0.75 ± 0.24	4.71 ± 0.54	5.57 ± 0.68
	III	0.17 ± 0.03	1.12 ± 0.21	7.22 ± 0.86	8.51 ± 0.95
	IV	0.18 ± 0.04	1.24 ± 0.37	11.31 ± 1.41	12.73 ± 1.46
	V	0.18 ± 0.06	1.27 ± 0.43	15.88 ± 2.51	17.33 ± 2.89
Carbon storage (kg culm^−1^)	I	0.04 ± 0.02	0.23 ± 0.07	1.30 ± 0.20	1.58 ± 0.23
	II	0.05 ± 0.02	0.36 ± 0.11	2.18 ± 0.25	2.59 ± 0.32
	III	0.08 ± 0.02	0.54 ± 0.10	3.34 ± 0.40	3.96 ± 0.44
	IV	0.08 ± 0.02	0.60 ± 0.18	5.23 ± 0.65	5.91 ± 0.68
	V	0.08 ± 0.03	0.61 ± 0.21	7.35 ± 1.16	8.04 ± 1.34

^a^Mean ± standard deviation

The initial stage of growth ended after about 40 days when the bamboo culms reached their maximum height and showed astonishing productivity, as shown in Table 3, where the aboveground biomass and carbon storage are 3.44–17.33 and 1.58–8.04 kg culm^−1^ for moso bamboo, respectively. Comparing the trends between biomass components, I found that all biomass components increase with increasing DBH class, especially the culm biomass increasing rapidly.

The structure of bamboo is such that main culms occupy over 80 % of aboveground biomass (Yen et al. 2010; Yen and Lee 2011). In this study, the proportions of foliage, branches and culms to total aboveground biomass were predicted to be 1–3, 7–14 and 83–92 %, respectively (Fig. 5). Moreover, that the proportions of foliage and branches decrease with increasing DBH class and that the proportion of culm increases with increasing DBH class are obviously shown in Fig. 5. This phenomenon may result from the fact that foliage biomass only occupies a small proportion of total aboveground biomass and that foliage biomass increases more slowly than other components with increasing DBH class.

**Fig. 6:**
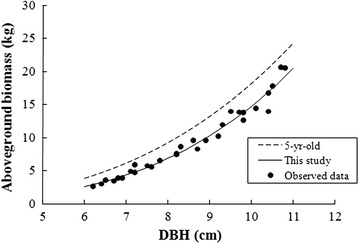
Relationships between aboveground biomass of observed data and DBH of moso bamboo and the allometric model were used to predict the biomass. The allometric model is Y = 0.0054DBH3.44 (*RMSE* = 0.8685 m, *R* ^*2*^ = 0.97) for this study. In addition, the allometric model of a 5-year-old (Y = 0.0171 DBH ^3.03^) was cited from Yen and Lee (2011) and was used for comparison in this study

In addition, the allometric model was used to predict the relationships between aboveground biomass and DBH for moso bamboo, and the results are shown in Table 4 and Fig. 6.

**Table 4:** The parameters of allometric models for predicting aboveground biomass of moso bamboo by DBH

Items	*Y* = *a* × *DBH* ^*b*^			
	a	b	*R* ^*2*^	*RMSE* ^*a*^
Initial stage (this study)	5.43 × 10^−3^	3.44	0.97	0.869
5-year-old^b^	1.71 × 10^−2^	3.03	0.96	2.098

^a^ *RMSE* is the smallest root mean squared error and its unit is m

^b^The 5-year-old bamboo model was cited from Yen and Lee (2011)

The allometric model of a 5-year-old plant, which was built by Yen and Lee (2011) in the same study area, was cited for comparison in this study. The allometric model fit the observed data well, with a small *RMSE* (0.869 m) and high *R* ^*2*^ (0.97). The observed data also closely matches the theoretical curve (Fig. 6). The observed data of aboveground biomass was compared to the allometric function of a 5-year-old plant, revealing that the ranges of ratios are from 0.61 to 0.92 and the average is 0.76. This indicates that the contribution of total biomass accumulation during the entire growth period (5 years) is 76 % for the initial stage.

## Discussion

This study focused on the development of bamboo during the initial growth stage and measured the culm height growth, biomass accumulation and carbon storage for different DBH classes. The development types of bamboo plantations are based on horizontal rhizomes, and the culms are sprouted by horizontal rhizome systems. Individual bamboos are propagated year by year; therefore, culms of different ages are distributed within the plantations and bamboo forests, which become even-aged stands (Yen et al. 2010; Yen and Lee 2011). For individual bamboo plants, development can be classified into two major stages (Lu 2001; Fu 2000; Yen 2003). The first stage lasts from bamboo shoot emergence from the ground to culms reaching their maximum height, and the second stage lasts from after the culms reach maximum height to when they are mature and can be harvested for human use (Lu 2001; Yen 2003; Yen and Lee 2011). The length of the first stage is usually only 40–60 days, while the second stage lasts about 4–5 years (Lu 2001; Yen 2003; Yen and Lee 2011). Comparing these two stages, the former occupies a much shorter time than the latter. In addition, the properties of bamboo reveal that the culms increase in strength and accumulate biomass in the second stage, but their height and DBH no longer increase (Yen and Lee 2011).

As the bamboo forests are an even-aged stand, the procedures for determining culm ages for the individual bamboos are important for predicting biomass or carbon storage in the whole stand. Moreover, predictions of the biomass or carbon storage are usually based on the allometric model, and this method has been widely applied to bamboo forests worldwide, such as *Bambusa bambos* (Shanmughavel et al. 2001), makino bamboo (Yen et al. 2010) and moso bamboo (Chen et al. 2009; Yen and Lee 2011). I also found that the properties of bamboo biomass have been widely researched during the second stage, but they have rarely been addressed during the first stage. The motives of the present study are to evaluate biomass accumulation and carbon storage and to predict culm development during the first stage for moso bamboo to understand the contributions of this period to the entire growing period.

The culm height growth of bamboo is rapid and usually uses days as the unit of measurement (Lu 2001; Yen 2003). The culm height growth of the observed data showed a sigmoid shape for moso bamboo for different DBH classes in this study (Fig. 2), a phenomenon that has also been found in a previous study (Yen 2003). The Richards function with a sigmoid shape is a famous model that has been widely applied in different fields, such as bamboo height growth (Yen 2003), timber tree growth (Colbert et al. 2004), tree height development (Christian and Oscar 2006), stand development (Pienarr and Turnbull 1973; Huuskonen and Miina 2007) and site index curves (Negash and Hubert 2006; Louw and Scholes 2006). In culm height development, Yen (2003) compared the three growth models, namely the Richards model, the Schumacher model and the Mitscherlich model, for moso bamboo in thinning and fertilization trials and found that the Richards model is superior to the others. Therefore, I directly selected the Richards model to predict the culm growth processes during the first stage and found an accurately simulated effect for all DBH classes. This indicated that the Richards function is suitable for the prediction of moso bamboo culm growth.

In addition, a regular growth pattern was revealed in culm height development for the different DBH classes based on the Richards function. Culm height growth can thus be predicted through the parameters of the Richards function. The parameters of the Richards function have special definitions. Namely, the *A* parameter implies growth potential, and the *k* and *m* parameters will affect the curve shapes of the Richards function (Richards 1959; Pienarr and Turnbull 1973; Yen 2003). In this study, I found that the *A* parameter ranges from 10.46 to 15.66 and increases with increasing DBH class, indicating that the growth potential of culm height increases with increasing DBH. Reasonably, a larger DBH has a higher growth potential of culm height for moso bamboo, and it can be predicted through the *A* parameter. However, the *k* and *m* parameters appear similar for different DBH classes, where the *k* and *m* parameters ranges are 0.0980–0.1006 and 0.8490–0.8664, respectively (Table 2). The *t*_*max*_ can be calculated through these two parameters as–*ln*(1−*m*)/*k* (Richards 1959; Pienarr and Turnbull 1973; Yen 2003). According to this formula, *t*_*max*_ is calculated to be 20.0, 18.8, 20.5, 20.0 and 19.5 days for DBH classes I to V, respectively. It was shown that the *t*_*max*_ is close to 20 days regardless of the DBH class. A similar result was discovered in the thinning and fertilization trials of Yen (2003), where the maximum culm growth rate also occurred at 20 days regardless of thinning and fertilization. In general, the growth patterns of culm heights may be influenced following different factors while the *t*_*max*_ appears to not be influenced.

The determination of biomass and carbon storage for woody plants became an import task after the signing of the Kyoto Protocol (Lamolom and Savidge 2003; Yen et al. 2009; Yen and Lee 2011). Bamboos can accumulate dry mass in a short period of time, and an astonishing accumulation capability has been found in certain bamboo species, especially under good management (Shanmughavel and Francis 1996; Isagi et al. 1997; Yen et al. 2009, 2010; Yen and Lee 2011).

The individual bamboo plants, from bamboo shoots to mature culms, only need about 4–5 years to grow (Lu 2001; Yen et al. 2010; Yen and Lee 2011). Therefore, the culms can be harvested after only 5 years. As bamboo forests are even-aged forests with 1- to 5-year-old culms within each plantation, one-fifth of the total culms can be harvested per year. If the bamboo plantations are under good management and each age culm has an average distribution, then the yields will be equal to the oldest culms. This is an ideal condition for the bamboo yield models. Yen and Lee (2011) used this model and predicted the yields of biomass and carbon storage to be 17.74 and 8.13 Mg yr^−1^ ha^−1^, respectively. Moreover, Yen and Lee (2011) compared the carbon sequestration between moso bamboo and China fir and found that moso bamboo was superior to China fir by a factor of 2.39.

From the viewpoint of carbon storage, many studies have revealed that bamboo can accumulate a high biomass during the growth period, with a high potential for carbon storage (Chen et al. 2009; Nath et al. 2009; Yen et al. 2009; Yen and Lee 2011; Yen and Wang 2013; Wu et al. 2015). Although a high productivity was discovered during the growth periods for bamboo, Yen and Lee (2011) pointed out that there was no significant accumulation of biomass in 1- to 5-year-old culms. This indicates that the highest contribution to biomass accumulation occurred during the first growth stage. The present study attempted to explore the contributions of biomass accumulations during the first stage for individual moso bamboo plants and found that the culms reached their maximum height at approximately 40 days, meaning the first stage is equal to 40 days. If the total yield period is 5 years (5 year × 365 days year^−1^=1825 days), then the first stage only occupies 2.2 % of the total yield period (5 years). However, during this period, the aboveground biomass and carbon storage are approximately 3.44–17.33  and 1.58–8.04 kg culm^−1^, respectively. I used the allometric model of a 5-year-old plant (the model was built by Yen and Lee 2011) to compare the observed data during the first stage. The mean was equal to 0.76 for biomass accumulation, implying that nearly three-fourths of total biomass accumulation occurs during the initial stage. Moreover, as mentioned above, biomass and carbon storage are highly correlated, and in general, carbon storage can be obtained via the biomass × PCC. As the carbon storage was calculated from biomass, I inferred that the ratio of the initial stage to the total growth period for carbon storage was still near three-fourths and this step was not further derived.

The biomass components of different sections to aboveground biomass showed 1–3, 7–14 and 83–92 % for foliage, branches and culms, respectively, for the initial stage of moso bamboo. Moreover, only that the proportion of culm increases with increasing DBH class while that the proportions of the other components decrease with increasing DBH class (Fig. 5). Yen (2003) pointed out that the foliage and branch developments begin in the initial stage of moso bamboo and their developments will continue to the second stage. I found a previous study of Yen and Lee (2011) and the present study with same site. The DBH range of their samples (6–11 cm) was also similar to the present study. Because a same condition of bamboo plantations was found between their study and the present study, I adopted the results of their study as the second stage to compare with the present study for moso bamboo. Yen and Lee (2011) determined the proportion of foliage, branches and culms to aboveground biomass for 1- to 5-year-old moso bamboo, and the proportions revealed 3–5, 12–17 and 83–85 % for foliage, branches and culms, respectively. In addition, the proportions of each component appear similar among different age classes (Yen and Lee 2011). Comparing 1- to 5-year-old moso bamboo plants with the plants in this study, the proportions of foliage and branches of this study are lower than that of 1- to 5-year-old moso bamboo, while the proportion of culms is higher than 1- to 5-year-old moso bamboo. This is because of the lower proportions of foliage and branches during the initial stage for moso bamboo.

Although moso bamboo accumulates three-fourths of its biomass for the entire growth period in only 40 days, I must emphasize that the culms still appeared weak. They cannot be used at that stage and should increase in strength and accumulate biomass for 5 years before being harvested for use. Moreover, the allometric model developed in this study was based on the regional data of the initial stage of moso bamboo. Therefore, this model might be not suitable for extending to moso bamboo plantations of the entire Taiwan. It also cannot be applied to predict the biomass of the second stage for moso bamboo.

## Conclusions

Many researches have addressed the ability of biomass accumulation for bamboo plants but little on comparisons of that of different stages. The present study compared the biomass accumulation between this stage and the entire yield period (5 years) and found that the bamboos accumulated three-fourths of their biomass for the entire yield period in only 40 days. This revealed that biomass accumulation and carbon storage mainly occurs in the initial growth stage for individual moso bamboos.
